# The relevance and applicability of oocyst prevalence as a read-out for mosquito feeding assays

**DOI:** 10.1038/srep03418

**Published:** 2013-12-04

**Authors:** Will J. R. Stone, Maarten Eldering, Geert-Jan van Gemert, Kjerstin H. W. Lanke, Lynn Grignard, Marga G. van de Vegte-Bolmer, Rianne Siebelink-Stoter, Wouter Graumans, Will F. G. Roeffen, Chris J. Drakeley, Robert W. Sauerwein, Teun Bousema

**Affiliations:** 1 Department of Medical Microbiology, Radboud university medical center, Nijmegen, The Netherlands; 2 Department of Immunology and Infection, London School of Hygiene and Tropical Medicine, London, United Kingdom; 3 These authors contributed equally to this work.; 4 Current address: Teun Bousema, Department of Medical Microbiology, Radboud university medical center, Geert Grooteplein 10, Nijmegen, 6525 GA

## Abstract

Mosquito feeding assays are important in evaluations of malaria transmission-reducing interventions. The proportion of mosquitoes with midgut oocysts is commonly used as an outcome measure, but in natural low intensity infections the effect of oocyst non-rupture on mosquito infectivity is unclear. By identifying ruptured as well as intact oocysts, we show that in low intensity *P. falciparum* infections i) 66.7–96.7% of infected mosquitoes experienced oocyst rupture between 11–21 days post-infection, ii) oocyst rupture led invariably to sporozoite release, iii) oocyst rupture led to salivary gland infections in 97.8% of mosquitoes, and iv) 1250 (IQR 313-2400) salivary gland sporozoites were found per ruptured oocyst. These data show that infectivity can be predicted with reasonable certainty from oocyst prevalence in low intensity infections. High throughput methods for detecting infection in whole mosquitoes showed that 18s PCR but not circumsporozoite ELISA gave a reliable approximation of mosquito infection rates on day 7 post-infection.

Malaria transmission-reducing drugs and vaccines are integral elements of the research agenda for malaria eradication^1^. Progression to a stage where these can be evaluated in clinical and community trials will be dependent on the ability of researchers to determine the infectivity of malaria exposed populations to anopheline vectors of *Plasmodium spp.*^2^,^3^. This requires mosquito feeding assays (MFA), wherein groups of mosquitoes are fed the blood of potentially infective individuals and subsequently examined to confirm the establishment of infection. In preparation for trials with transmission reducing interventions (TRI), efforts are being made to standardise MFA^4^,^5^,^6^,^7^,^8^,^9^. Because of the laboriousness of MFAs and the necessity of large numbers of mosquito observations for precise estimates^8^, increasing the scalability of MFA evaluation has been identified as a research priority^5^,^6^.

The scalability of MFA is to an important extent defined by the chosen endpoint. During sporogonic development, ingested male and female *Plasmodium* gametocytes quickly activate to form gametes in the mosquito gut. Ookinetes develop from the fusing of gametes, and penetrate the midgut wall forming oocysts under the basal lamina of the midgut epithelium. Upon oocyst rupture, sporozoites are released that migrate to mosquito salivary glands rendering the mosquito infective.

In the context of public health the most important measure of TRI efficacy is a reduction in the number of mosquitoes that become infective after feeding on a treated or vaccinated individual^2^. Salivary gland invasion can be detected around day 14 post infection (PI). In MFA, examining mosquitoes at an earlier time-point has advantages for mosquito husbandry, improves the likelihood of mosquito survivorship until examination, and avoids the direct health hazard that infective mosquitoes form for laboratory personnel. The presence or intensity of oocysts observed on mosquito midguts, which for *P. falciparum* can be reliably detected by microscopy as early as day 6 PI^6^, is therefore commonly used as an operationally attractive outcome measure that may also be amendable for high throughput processing by immunological or molecular methods. However, this requires that the detection of any number of oocysts should reasonably predict their likelihood of causing mosquito infectivity^2^. At high oocyst intensities such as are achieved with cultured gametocytes in the standard membrane feeding assay (SMFA)^8^,^9^,^10^, the progression from oocyst formation to mosquito infectivity is assumed. In MFA on naturally infected individuals, oocyst intensities within the range 1 to 5 are commonly observed^11^,^12^,^13^, and it is currently not fully understood how these low oocyst densities relate to later sporozoite development^8^. Experiments that simultaneously detected intact oocysts and salivary gland sporozoites or compared oocysts and sporozoites in separate groups of mosquitoes suggest that not all oocysts contribute effectively to mosquito infectivity by releasing sporozoites into the haemocoel^14^,^15^,^16^,^17^. The impact of oocyst arrest in low intensity infections might decrease the reliability of oocyst prevalence as an indicator of infectivity because it could lead to the total failure of sporozoite release.

To validate the development of whole-mosquito approaches to infection detection, we report the results of experimental assessments of the dynamics of *P. falciparum* oocyst-sporozoite development. Using antibody staining techniques we directly determine the extent of oocyst rupture in low intensity mosquito infections and in the same mosquitoes, assess the efficiency of sporozoite production or invasion of the salivary glands. The suitability of mosquito infection detection by both circumsporozoite protein (CSP) ELISA and *P. falciparum* specific 18s PCR was determined in comparison with routine microscopy.

## Results

### Achieving low oocyst intensity infections

**Figure 1:**
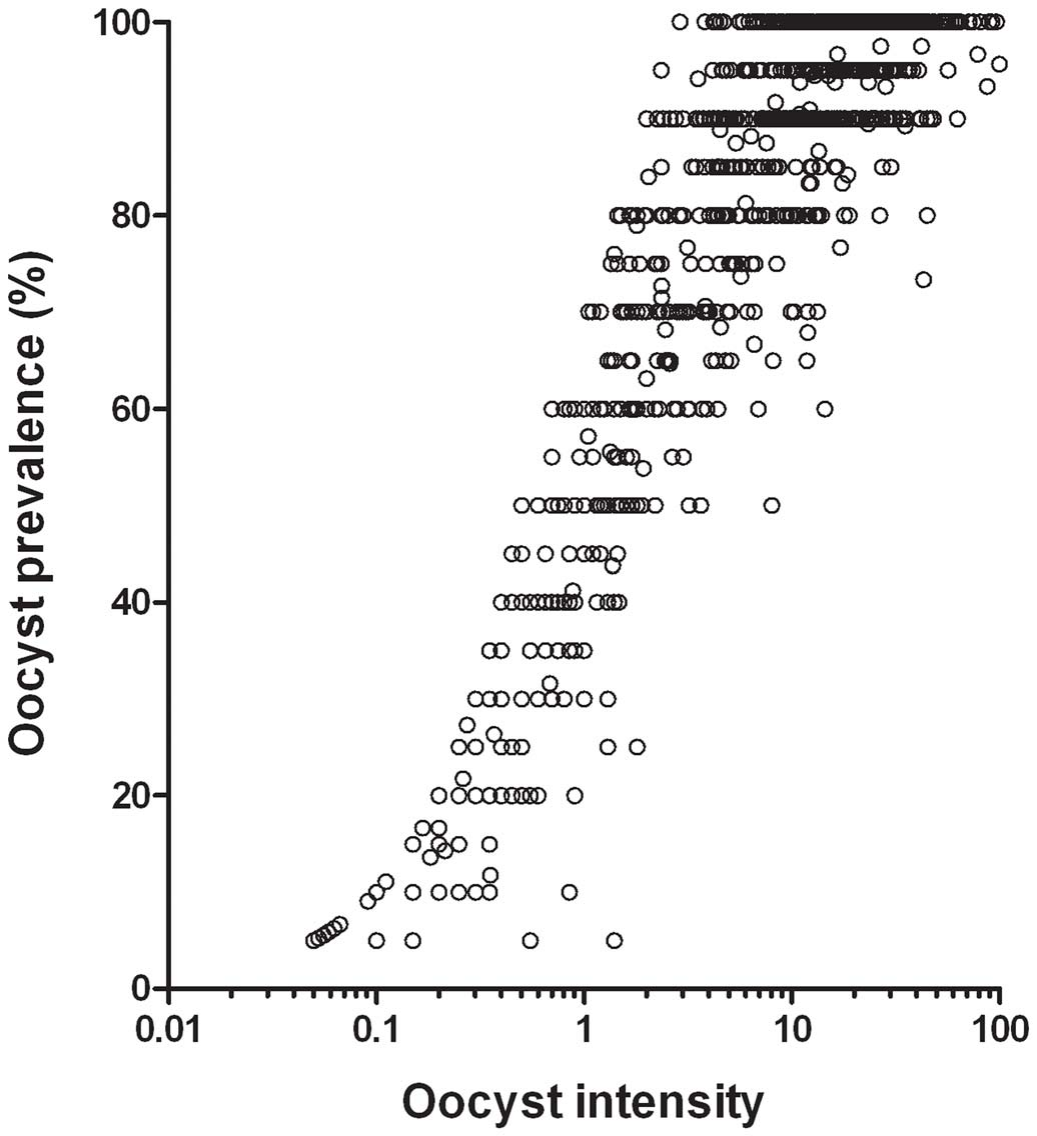
Oocyst prevalence and intensity for experimental feeds utilising the NF54 *P. falciparum* strain conducted in the mosquito infectivity unit at RUMC, Nijmegen between 2011 and 2013. Each data point represents the mean oocyst intensity and oocyst prevalence in a single group of mosquitoes. Criteria for inclusion in these figures were sample size (>10 mosquitoes), parasite strain (NF54 strain only) and mosquito species. Data were collected from both experimental and control feeds between 6 and 9 days PI. In total 1323 separate experimental feeds comprising 21240 mosquito dissections are shown (1233 experimental feeds with *A. stephensi*, 90 experimental feeds with *A. gambiae*).

In line with previous observations^8^,^18^ oocyst prevalence and intensity were highly related (Fig. 1). Gametocyte culture material was diluted with uninfected blood to achieve oocyst intensities that are in line with the 1–5 oocysts typically observed in natural *P. falciparum* infections. Infection prevalence (measured at day 7 PI) for the separate experiments (n = 30–104) ranged between 33% and 86.5%, with mean oocyst intensities from 0.57 and 4.7. In total 1591 mosquitoes were used in the current study (405 *A. stephensi*, 1186 *A. gambiae*). Overall median oocyst density in infected mosquitoes was 2 oocysts (IQR 1–5, range 1–10).

### Measuring oocyst rupture in low intensity infections

**Figure 2:**
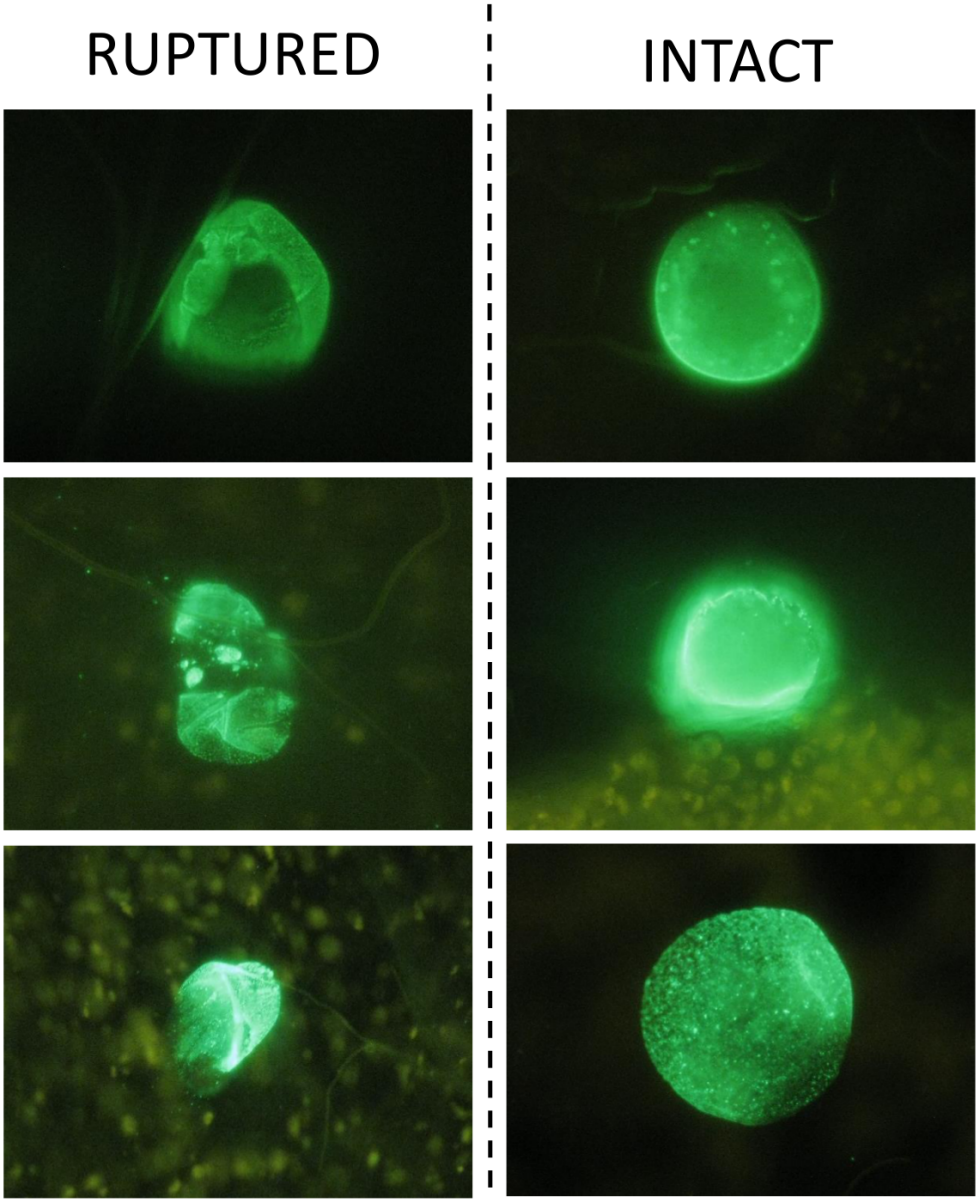
Classification of oocyst condition. Mosquito midguts were dissected in phosphate buffered saline (PBS) and stained using 3SP2-Alexa488 anti-CSP antibodies. After staining, midguts were washed twice with PBS for 10 minutes before being sealed under a glass cover slip with Vaseline petroleum jelly. All oocysts were identified and assigned a condition by two independent microscopists. Intact oocysts were visibly unbroken. Ruptured oocysts and oocyst remnants were visibly broken or degraded. All samples were examined within 8 hours of dissection, but sample condition remained stable for a number of days (stored at 4°C) prior to dissection for re-examination. Three ruptured and three intact oocysts are shown in the figure for demonstrative purposes.

**Figure 3:**
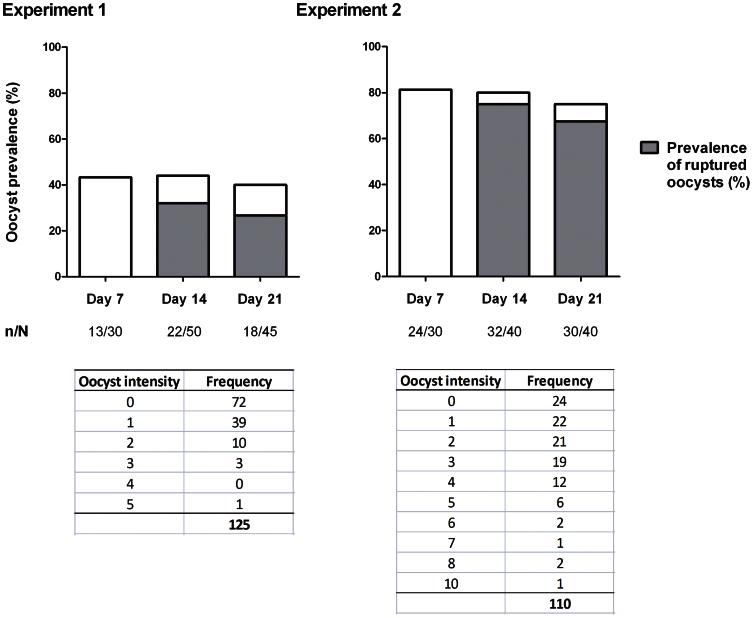
Total oocyst prevalence and prevalence of oocyst rupture. n/N = oocyst positive mosquitoes/total number of mosquitoes dissected. Prevalence of oocyst rupture is given as the proportion of mosquitoes in which any oocysts were observed to have undergone rupture. Tables indicate the range and frequency of oocyst intensities observed in experiment 1 and 2. In experiment 1, 16/22 (72.7%, 95% CI = 49.8–89.3%) infected mosquitoes had at least one ruptured oocyst at day 14 PI, while 12/18 (66.7%, 95% CI = 41–86.7%) were rupture positive at day 21 PI. In experiment 2, 30/32 (93.8%, 95% CI 79.2–99.2%) infected mosquitoes were rupture positive at day 14 PI, while 27/30 (90%, 95% CI = 73.5–97.9%) were rupture positive at day 21 PI.

To determine the extent of oocyst rupture in mosquitoes with a low oocyst intensity, intact oocysts were detected among sub-groups of a single cage of *A. stephensi* at days 7 (n = 20), 15 (n = 20), 17 (n = 20), 21 (n = 20), 24 (n = 20) and 27 (n = 20) PI. The prevalence of intact oocysts on days 7, 15, 17, 21, 24 and 27 was 100% (95% CI 96.4–100%), 45% (95% CI 23.1–68.5%), 25% (95% CI 8.7–49.1%), 25% (95% CI 8.7–49.1%), 40% (95% CI 19.1–63.9%) and 20% (95% CI 5.7–43.7%) respectively (see Fig. S1 in the Supplementary information online). Four experiments were subsequently conducted using antibody staining to identify ruptured as well as intact oocysts (Fig. 2). In the largest two experiments, oocyst prevalence and median intensity were first determined in groups of 30 mosquitoes by standard mercurochrome staining at day 7 PI, before a total of a total of 175 mosquitoes were dissected for antibody staining at days 14 and 21 PI (Fig. 3). For experiment 1, oocyst prevalence at day 7 PI was 43%, and median oocyst intensity in infected mosquitoes was 1 (IQR 1–2, range 1–5). In experiment 2, oocyst prevalence at day 7 was 80%, while median oocyst intensity for infected mosquitoes was 3 (IQR 1–4.3, range 1–10). No significant differences were observed in ruptured oocyst prevalence (the proportion of mosquitoes with ≥1 ruptured oocyst) or mean intensity between mosquito groups dissected at day 14 or 21 PI, indicating that oocyst rupture after day 14 PI was limited. Rupture and oocyst prevalence at all time points are given in Fig. 3. In the two smaller experiments (3&4) oocyst prevalence and median intensity were first determined in groups of 30 mosquitoes by standard mercurochrome staining at day 7 PI and oocyst rupture was subsequently determined between 11–16 days PI, reserving the mosquito carcass for assessments of sporozoite intensity. For experiment 3 (n = 98), median oocyst intensity in infected mosquitoes was 2 (IQR 1–4, range 1–10), oocyst prevalence at day 7 was 80%, and the proportion of oocyst positive mosquitoes with at least one ruptured oocyst was 84.6% (66/78, 95% CI 74.7–91.8%). For experiment 4 (n = 39), median oocyst intensity in infected mosquitoes was 2 (IQR 1–3, range 1–9), oocyst prevalence at day 7 was 77%, and the proportion of oocyst positive mosquitoes with at least one ruptured oocyst was 96.7% (29/30, 95% CI 82.8–99.9%). The prevalence of oocyst rupture within oocyst positive mosquitoes for experiments 1–4 is shown as Supplementary Fig. S2 online.

Taken together, our results show that in mosquitoes where the majority of infections stemmed from 1–3 oocysts, 72% (390/542) of all oocysts had undergone rupture at the time of observation, while 66.7–96.7% of infected mosquitoes had at least one ruptured oocyst.

### Sporozoite production and invasion of the salivary glands are uniformly effective in mosquitoes with low oocyst intensities

To investigate the efficiency of sporozoite production from ruptured oocysts, the (qualitative) presence of sporozoites released into the haemocoel was determined both by microscopy after antibody staining and by PCR in mosquitoes which had undergone midgut dissection for oocyst detection. By removing the mosquito midgut for oocyst detection, sporozoite detection was restricted to those released from the midgut into the mosquito body. Sporozoites were detected in all rupture positive mosquitoes by both microscopy and PCR (85/85). No sporozoites were detected in mosquitoes without evidence of rupture (0/17).

**Figure 4:**
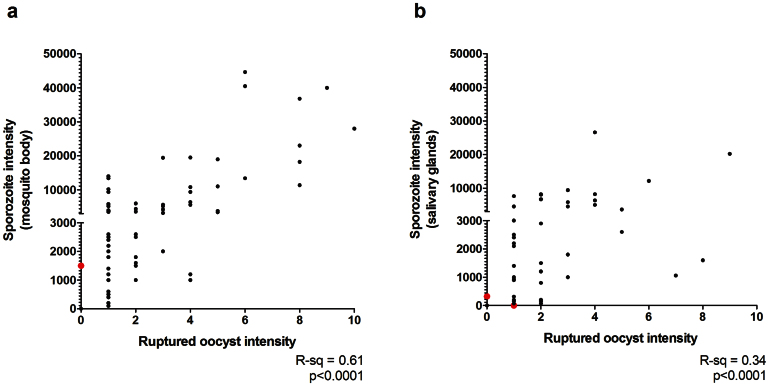
The number of sporozoites in the mosquito body or salivary glands, and ruptured oocyst intensity in the same mosquitoes. The detection limit for sporozoites detected in a single mosquito sample was 78 sporozoites (1 sporozoite observed in 64 Bürker chamber fields/0.256 μl homogenate). The Y-axis is split from 1–3000 sporozoites and from 3000–50,000 sporozoites because of the heterogeneous distribution of sporozoites within mosquitoes. Data points in red indicate erroneous observations (sporozoite +, rupture – or *vice versa*). a. Y-axis: Sporozoite total calculated from duplicate samples of whole homogenised mosquito (excluding gut) stained with 3SP2-Alexa488 conjugate antibodies. X-axis: Ruptured oocyst intensity. Median oocyst intensity in infected mosquitoes was 2 (IQR 1–4, range 1–10). The data point in red is a single mosquito for which 1500 sporozoites were found associated with no ruptured oocysts. b. Y-axis: Sporozoite total calculated from duplicate samples of homogenised 3SP2-Alexa488 stained salivary glands only. X-axis: Ruptured oocyst intensity. Median oocyst intensity in infected mosquitoes was 2 (IQR 1–3, range 1–9). The data points in red are a mosquito in which 320 sporozoites were found associated with no ruptured oocysts, and a mosquito in which a single ruptured oocyst was found associated with no sporozoites.

To examine the efficiency of sporozoite production and invasion of the salivary glands, groups of mosquitoes were examined for the detection of ruptured oocysts and quantification of sporozoites within the mosquito body (excluding the gut) (Fig. 4a) and the salivary glands (Fig. 4b). In mosquitoes in which sporozoites were detected in the body, a positive correlation between ruptured oocyst number and sporozoite intensity was observed (R^2^ = 0.61, p < 0.0001). Furthermore, all mosquitoes with detectable oocyst rupture were sporozoite positive, indicating that rupture will always result in a release of sporozoites into the mosquito haemocoel. In a single mosquito sporozoites were detected while no ruptured oocysts were detected by microscopy. It is probable in this instance that a ruptured oocyst was mistakenly identified as being intact. Considering all mosquitoes with ruptured oocysts, sporozoite intensity in the mosquito body ranged from 100–44600. Median production was 2000 sporozoites/ruptured oocyst (inter-quartile range (IQR) 950–3500). When considering only those cases where a single oocyst was found to have ruptured, the median number of sporozoites observed per oocyst was 2100 (IQR 1000–3750).

Among the 66 mosquitoes which underwent salivary gland dissection, 45 out of 46 rupture positive mosquitoes were also gland positive (97.8%, 95% CI = 88.6–99.9%) (Fig. 4b). In another mosquito, 320 sporozoites were found while no ruptured oocysts were detected. The relationship between the intensity of ruptured oocysts and sporozoite intensity in the salivary glands was positive, but as for sporozoites in the mosquito body, not clearly linear (R^2^ = 0.34, p < 0.0001). Salivary gland sporozoite intensity ranged from 78–26600. The median number of sporozoites in the glands was 1250 per oocyst (IQR 313–2400). For single ruptured oocysts only median sporozoite intensity per oocyst was 1000 (IQR 256.5–2450).

### The application of oocyst prevalence as a high throughput read-out system

Having demonstrated that at the lowest levels of oocyst intensity oocyst rupture and sporozoite release occurs in the majority of mosquitoes, we sought to determine the utility of high throughput techniques for the detection of any mosquito infection at time points after which oocyst development may have occurred. Nine experimental feeds were conducted. At day 7 PI, 30 mosquitoes were dissected as standard for oocyst detection. Separate groups of mosquitoes were then taken from the same cages at days 7, 10 and 14 PI for individual homogenisation. Mosquito homogenate was processed by both CSP ELISA and 18s PCR (Table 1). Full details of these experiments are given in Supplementary Table S1 online. In total 796 mosquitoes were tested by both CSP ELISA and 18s PCR. When compared to microscopy, ELISA underestimated mosquito infection rates in 100.0% (8/8) of experiments when ELISA was performed on day 7 and in 11.1% (1/9) of experiments when ELISA was performed on day 10. When ELISA was performed on day 14, there was no underestimation of infection rates compared to microscopy and 22.2% (2/9) of experiments had higher mosquito infection estimates by ELISA compared to microscopy (Table 1). By contrast, mosquito infection prevalence at day 7 determined by 18s PCR and microscopy did not significantly differ in any of the 8 experiments (experiment 8, p = 0.054), while at day 10 and 14 PI PCR prevalence was significantly higher than microscopy prevalence in 1 and 2 experiments respectively. In the two day 14 experiments where prevalence was higher by both ELISA and 18s PCR than by microscopy, differences in paired ELISA and PCR measures were non-significant or borderline significant (Day 14 Experiment 7, p = 0.046; Day 14, Experiment 8 p = 0.317).

Relative to positivity by PCR, the sensitivity of CSP ELISA for the detection of *P. falciparum* in whole homogenised mosquitoes increased from 6.8% at day 7 PI to 61.7% at day 10, and 86.7% at day 14 PI. 28/796 mosquitoes (3.5%) were determined as positive for CSP but negative for *P. falciparum* by PCR. ELISA OD values for these mosquitoes varied widely above the cut off for positivity of OD 0.12 (median OD (IQR) = 0.336 (0.14–0.91). Of the 256 mosquitoes determined negative by ELISA but positive by PCR (32.2% of total), 64.1% (164/256) were mosquitoes collected on day 7 PI, and 28.5% (73/256) were collected at day 10 PI. Agreement values between ELISA and PCR were 40.0% at day 7 PI (Kappa 0.02, p = 0.15), 71.2% at day 10 PI (Kappa 0.45, p > 0.0001) and 85.3% at day 14 PI (Kappa 0.69, p > 0.0001). Between paired measures positivity rates by PCR and ELISA were significantly different at day 7 (p < 0.0001) and 10 PI (p < 0.0001) but not at day 14 PI (p = 0.49).

## Discussion

The proportion of mosquitoes that become infective is the most relevant outcome measure for assessments of the human infectious reservoir for malaria and for evaluating the impact of transmission reducing interventions on human infectivity. If the extent of oocyst non-rupture in mosquitoes with low oocyst intensities is not prohibitively high, and if sporozoite release and invasion of the salivary glands is effective, then the detection of oocysts, regardless of their density, provides an acceptable estimate of mosquito infectivity for high throughput assessment. In this study we show that the majority of low density infections result in oocyst rupture, sporozoite release and colonisation of the mosquito salivary glands. We further show that PCR but not CSP ELISA may replace microscopy without a loss in sensitivity when examining mosquitoes on day 7 post infection.

The first aim of the current study was to establish the usefulness of oocyst prevalence as a stand-alone indicator of infectivity for the evaluation of MFA. The transition from oocyst formation to sporozoite transmission to humans was recently highlighted by Churcher *et al.* as a major gap in our current understanding and limitation to the to the evaluation of TRI^8^. The valuable few studies which have investigated the dynamics of late sporogony are summarised by Vaughn *et al.*^17^. A common observation among these studies is the presence of intact oocysts when most others have ruptured^14^,^15^,^16^. Rates of oocyst arrest have only been recorded in detail for *P. vivax* by Zollner *et al.,* who estimated that sporozoite production reached only 11% of its theoretical potential by day 18 PI, due to sub-optimal productivity by some oocysts or their total failure to release sporozoites^15^. Though non-rupture of a proportion of oocysts is not necessarily relevant where oocyst intensity is high, its occurrence in low intensity infections may significantly affect the accuracy of infectivity estimates based on oocyst prevalence alone. By visualising ruptured oocysts, we provide direct evidence that in low density infections (median oocysts in infected mosquitoes = 2, IQR 1–5) rupture occurs in the majority of *P. falciparum* infections. Combining the results of 4 independent experiments, 85.7% (180/210) of mosquitoes with low density infections had at least one ruptured oocyst when measured between 11–21 days post infection. Our data support previous estimates of the efficiency of sporozoite production and salivary gland invasion by directly relating these measures to the intensity of oocyst rupture measured in the same mosquitoes, showing that infectivity after sporozoite release is almost uniformly effective. Following oocyst rupture, sporozoite production appears to be highly effective; 204 of the 205 mosquito's containing ruptured oocysts were also sporozoite positive outside the midgut. In the current study, gland invasion was achieved in 45/46 mosquitoes with evidence of oocyst rupture. The one instance where rupture was detected and salivary gland sporozoites were not may have arisen because sporozoites from this oocyst may not have successfully reached the salivary glands^14^,^16^, or because rupture occurred so close to the time of dissection that haemocoel traversal was ongoing^19^,^20^.

Previous estimates of the productivity of *Plasmodium* oocysts have been restricted either because estimates were carried out artificially (excising oocysts from midguts and counting the sporozoites within)^21^,^22^, or because they were based on the observation of intact oocysts without consideration of the proportion which might fail to rupture^14^,^15^,^16^,^23^. In the current study, our direct approach to oocyst quantification and classification allowed for more precise estimates of *per capita* potential and effective sporozoite production. For each ruptured oocyst a mean of 2000 sporozoites were found in the mosquito body while 1250 were observed in the salivary glands, suggesting that the efficiency of gland invasion is ~60% of the total number of sporozoites released. Had oocysts been considered irrespective of their condition, per oocyst sporozoite numbers would have been reduced by approximately 28%, to 1440 in the body and 900 in the salivary glands (72% of all oocysts that were observed on mosquito midguts ruptured). Encouragingly, these estimates very closely match previous observations in the same species combination (1361 salivary gland sporozoites per oocyst, based on data from 33 mosquitoes)^23^. The results of the current study indicate that though the overall efficiency of sporozoite production, release and traversal may be relatively low, the effectiveness is nonetheless extremely high with the vast majority of oocyst positive mosquitoes eventually becoming sporozoite positive. There is no obvious bottleneck preventing the establishment of salivary gland invasion where they are released.

As oocyst intensities in the current study were manipulated to exist over a very narrow range (>80% 1–3 oocysts, range 1–10), analysis of the relationship between rupture rates and oocyst intensity was not possible. Though it is likely that at high oocyst intensities competition for nutrients may inhibit rates of oocyst maturation and rupture^15^,^24^,^25^, we consider it unlikely over the range of oocyst intensities observed in the current study that rupture rates would have decreased significantly with oocyst intensity, especially since a second bloodmeal was given to increase availability of nutrients and better mimic natural mosquito feeding frequencies.

For evaluations where mosquito infections levels are naturally low, such as for studies that aim to determine the human infectious reservoir for malaria or determine the impact of transmission reducing interventions for which the mode of action is before oocyst formation (e.g. gametocytocidal drugs or pre-fertilization and anti-ookinete transmission-blocking vaccines), we consider oocyst prevalence a reliable, albeit not perfect, indicator of mosquito infectiveness. Since the majority of oocyst positive mosquitoes had at least one ruptured oocyst from which sporozoites reached the salivary gland, and as the number of sporozoites egested during probing (<100) is considerably less than the number produced by single oocysts (1,359–14,000)^21^,^22^,^26^,^27^,^28^ and independent of total salivary gland sporozoite intensity^28^, we consider it plausible that infectivity can be predicted with reasonable certainty from oocyst prevalence. The modest overestimation of infectivity that is a consequence of some oocysts not rupturing in low density infections would be acceptable for the evaluation of programs aiming to interrupt parasite transmission. However, this overestimation may require consideration in studies where direct effects on sporozoite development or invasion of the salivary glands may be responsible for variations in the proportion of mosquitoes that are sporozoite-positive^29^.

Using oocyst prevalence as the sole outcome measure for mosquito feeding assays has advantages in terms of mosquito husbandry, the number of mosquitoes surviving long enough to contribute to read out measures and laboratory safety. As such, the results of the current study open the way for the replacement of microscopy with high throughput techniques for oocyst detection to allow for the large number of mosquito observations needed to reliably estimate TRI efficacy^8^. We explored two scalable options: CSP-ELISA and 18s PCR. PCR and ELISA have previously been used for the detection of midgut oocysts^24^,^30^ or salivary gland sporozoites^31^,^32^,^33^,^34^ in dissected colony and wild caught mosquitoes. In the current study, the same homogenate from undissected mosquitoes was used for both PCR and CSP-ELISA. CSP-ELISA underestimated mosquito infection rates compared to microscopy and PCR, especially at day 7 PI when sporozoite production and antigen release from oocysts may have been limited^19^. Several approaches were explored for mosquito homogenisation and maximization of CSP release from midgut oocysts including sonication, rapid freeze-thaw cycling, and preparation with various detergents. None of these approaches solved the apparent lack of CSP release from PCR-detectable oocysts (data not shown) that precludes the use of CSP-ELISA as read-out for mosquito feeding assays before day 10 PI. In contrast, 18s PCR showed good agreement with microscopical oocyst prevalence at all time points. Where statistical differences in prevalence were observed these were because of higher prevalence by PCR, suggesting that microscopy may have missed a small number of (low density) infections. Day 14 CSP-ELISA results supported PCR estimates in experiments where PCR infection prevalence was higher than microscopy prevalence, suggesting that differences in oocyst prevalence between samples may also have affected these outcomes. The earliest time point at which the whole-mosquito approach to oocyst detection can be applied depends upon the rate at which non-established parasites are cleared from the digestive tract of mosquitoes following the infective blood meal. Bell and Ranford-Cartwright estimated using PCR based on the amplification of the MSA-1 gene that *Plasmodium* DNA can no longer be detected in the mosquito body ≥5 days PI^24^. Using the current technique, this creates a boundary at 5 days PI after which any *Plasmodium* in mosquitoes represents an established infection. Ookinete specific markers could bring forward this boundary, but to establish the utility of such markers the efficiency of ookinete-oocyst transition would require further investigation; current estimates indicate that in *Plasmodium* species affecting humans population loss during the ookinete-oocyst transition may be between 50–75%^17^.

The results of the current study validate the use of oocyst prevalence as an indicator of infectivity. Sensitive PCR based approaches to the detection of mosquito infection at time points after oocyst formation are capable of replicating the results of standard microscopical oocyst prevalence assessments.

## Methods

### Mosquito infections

#### i) Mosquito rearing and parasite culture

*Anopheles stephensi* (Sind-Kasur Nijmegen strain)^35^ and *A. gambiae* (Ngousso strain)^36^ were reared at 30°C and 70–80% humidity, while exposed to a 12/12 hour day/night cycle. Direct comparisons of SMFA experiments using *A. stephensi* and *A. gambiae* observed no differences between mosquito species in the association between oocyst prevalence and oocyst density or estimates of transmission reduction of transmission-blocking monoclonal antibodies or field sera (Eldering *et al.*, in preparation). For the current experiments, *A. stephensi* mosquitoes were used for all experiments on sporogonic development. The experiments on high-throughput assessment of infected mosquitoes by PCR or ELISA used *A. gambiae* mosquitoes to allow a direct comparison with field-based mosquito feeding assays. Mature *P. falciparum* (NF54) gametocytes (14 day culture, 0.3–0.5% gametocytes, 2% haematocrit) were obtained from an automated tipper system and prepared as previously described^37^,^38^. To achieve low intensity infections for examinations of oocyst condition and sporozoite production, infective blood meals that are routinely used and produce high infection prevalence (averaging >70% with oocyst intensities in infected mosquitoes >10) were diluted at a ratio of 1:10 with uninfected blood. Dilutions between 1:5 and 1:20 were used for comparisons of microscopy, ELISA and PCR for the detection of infection among groups of mosquitoes, where we aimed for a range of infection intensities (see Supplementary Table S1 online).

#### ii) Mosquito feeding and dissections

3–5 day old mosquitoes were fed on a glass membrane midi-feeder system containing ~1.25 ml of the *P. falciparum* culture mix^37^,^38^. Unfed and partially fed mosquitoes were removed after feeding and blood fed females were maintained at 26°C and 70–80% humidity. For examinations of oocyst condition and sporozoite production, mosquitoes received an additional uninfected human blood meal between 7 and 10 days PI^39^. Routine staining of midguts was done in 1% mercurochrome.

### Immunostaining experiments

#### i) Oocyst prevalence, rupture and sporozoite prevalence at different time-points post-infection

To examine oocyst condition and sporozoite prevalence at different time-points PI, the midguts of 30 *A. stephensi* mosquitoes were removed at day 7 PI, stained with mercurochrome, and microscopically examined for oocysts prior to rupture. At both days 14 and 21 PI, two groups of mosquitoes were dissected (175 observations total) for oocyst and sporozoite examination by antibody staining, ensuring that in each of the four replicates approximately 20 positive mosquitoes were examined. At these later time-points midguts were dissected in 20 μl phosphate buffered saline (PBS, pH 7.2), transferred to a fresh drop of PBS and stained using 3SP2-Alexa488 anti-CSP antibodies (1:400) for 30 minutes at room temperature (RT) in a humid container. Details of the 3SP2 antibodies used for oocyst/sporozoite staining and CSP ELISA (produced in Nijmegen, Netherlands) have been previously described^28^,^40^. After staining, midguts were washed twice with PBS for 10 minutes before being sealed under a glass cover slip with Vaseline petroleum jelly (Unilever, UK). During all dissection and washing steps care was taken to avoid artificial oocyst rupture. Intact/degenerated and ruptured oocysts were counted using an incident light fluorescence microscope. Mosquito carcasses and the PBS from the first dissection step were homogenised in glass grinding tubes with 180 μl of PBS. Two lots of 15 μl of homogenate were transferred to a glass slide, incubated with 5 μl of 3SP2-Alexa488 conjugate (1:400), and left for 30 minutes at RT in a humid container. After staining, cover slips were applied with Vaseline and sporozoite positivity was determined by examining 50 fields for each sample (100 fields per mosquito) using an incident light fluorescence microscope.

#### ii) The number of sporozoites released by ruptured oocysts

To estimate the contribution of ruptured oocysts to sporozoite intensity in the whole mosquito body, the midguts of 98 *A. stephensi* mosquitoes were dissected and stained between days 11–14 PI using 3SP2-Alexa488 antibodies as described above. Mosquitoes were collected across four feeding experiments, in which mosquito homogenate was transferred to counting chambers (Bürker, Labor Optik, UK) and allowed to settle for 20 minutes in a humid container before quantification was conducted using an incident light fluorescence microscope.

#### iii) The number of sporozoites invading the salivary glands following oocyst rupture

To determine the association between oocyst rupture and mosquito infectivity, the midguts and salivary glands of 66 *A. stephensi* mosquitoes were carefully dissected between days 12–16 PI. Mosquitoes were collected across two feeding experiments. Salivary glands were removed from the mosquito carcass and homogenised in glass grinding tubes with 20 μl of 3SP2-Alexa488 conjugate (1:400). Sporozoite quantification was then conducted as described for body sporozoites.

### High throughput mosquito processing and infection detection

#### i) Mosquito processing and experimental design

Nine cages of approximately 160 *A. gambiae* mosquitoes were infected as described above for investigation of the comparative efficacy of microscopy, ELISA and PCR in detecting mosquito infection prevalence. Alongside standard dissections carried out on a sample of mosquitoes at day 7 PI, CSP-ELISA and PCR were performed on each mosquito in groups collected from each cage at day 7, 10 and 14 PI. Details of sample sizes (n = 10–44) are in Table 1, and full details of all results are in Supplementary Table S1 online.

#### ii) CSP-ELISA

CSP ELISA was performed as previously described, with minor modifications^41^. Mosquitoes were homogenised in 250 μl phosphate buffered saline (PBS pH 7.2) solution with 1% sarcosil. Sterilin ELISA plates were coated with 3SP2 (Nijmegen, Netherlands) at 5 μg/ml, diluted in PBS. Homogenate of test mosquitoes were analysed in duplicate, alongside blank wells (just sample diluent) and a standard curve of recombinant CSP (Gennova, 0.1 μl/ml). A selection of homogenates from 30 uninfected blood-fed control mosquitoes were tested on every plate, so that each sample was tested at least 5 times over the course of the experiment. A cut off for CSP positivity was determined at an optical density (OD) of 0.12, which was the mean OD of the uninfected mosquitoes plus three standard deviations.

#### iii) DNA extraction and 18s PCR

Phenol-chloroform DNA extractions were carried out on the 150 μl of mosquito homogenate remaining from CSP ELISA as described previously, with minor modifications (23). Briefly, Proteinase K was added to each sample of mosquito homogenate at 20 mg/ml, and samples were incubated overnight at 55°C. After incubation, samples were briefly spun down and a Phenol/Chloroform/Isoamyl alcohol (25:24:1) mix was added to each sample at a ratio of 1:1. Samples were mixed vigorously and centrifuged at 10,000 g for five minutes. After spinning, the aqueous layer was mixed with 250 μl of Isopropanol, and 100 μl of a mix of 3 M Sodium Acetate (pH 5.2), Glycogen (10 mg/ml), and nuclease free water. Samples were then mixed repeatedly during a 15 minute incubation at RT, before centrifugation at 13,000 g for 30 minutes at 4°C. Supernatant was removed, washed with 1 ml of 70% Ethanol, and spun again for 5 minutes at 4°C. Ethanol was then removed, and the pellet dried and suspended in 20 μl of nuclease free water.

PCR targeting the small sub-unit ribosomal RNA (ssrRNA) gene of *P. falciparum* was performed as previously described, with minor modifications (24). The volume of DNA used in the nest 1 reaction was increased from 1 μl to 5 μl, and the volume of nest 1 template used in the nest 2 PCR was adjusted to 2 μl. For a more detailed overview of primer sequences, controls and PCR cycling conditions see Baidjoe et al. 2013^42^. N1 and N2 products were mixed and 10 μl was visualized on 1.5% agarose gel by electrophoresis in 0.5× Tris-acetate-EDTA buffer (0.04 M Tris-acetate and 1 mM EDTA, pH 8.0).

### Data analysis

Statistical analysis was conducted using STATA 12 (StataCorp., TX, USA) and GraphPad Prism 5.0 (GraphPad Software Inc., CA, USA). In the analyses of oocyst prevalence and condition, and between groups of mosquitoes analysed by dissection and PCR or ELISA, differences in prevalence between groups were tested by Chi-squared test. Differences between paired PCR and ELISA positivity values were tested by McNemars' Chi Square test. For agreement between PCR and ELISA carried out on the same mosquitoes, sensitivity, specificity, agreement and kappa values were presented using PCR for reference. Associations between ruptured oocyst intensity and sporozoite intensity were quantified with Spearmans correlation coefficients. *Per capita* sporozoite production (for both whole mosquito and salivary gland specific estimates) was presented as the mean of the sporozoite production per mosquito divided by the number of ruptured oocysts observed within the same mosquito.

## Supplementary Material

Supplementary Information Supplementary information

## Figures and Tables

**Table 1:** The results of nine experimental feeds where oocyst prevalence was determined in a sample of mosquitoes at day 7 PI, and infection prevalence was subsequently determined in separate samples collected 7, 10 and 14 days PI by both CSP ELISA and 18s PCR

Microscopy Prevalence % (n/N)			CSP ELISA Prevalence % (n/N)						18s PCR Prevalence % (n/N)					
Exp. ID	*Day 7 P1*		*Day 7 PI*		*Day 10 PI*		*Day 14 PI*		*Day 7 PI*		*Day 10 PI*		*Day 14 PI*	
**1**	63.3	(19/30)	2.5*	(1/40)	17.5*	(7/40)	53.8	(21/39)	66.7	(26/39)	60	(24/40)	64.1	(25/39)
**2**	36.7	(11/30)	0.0*	(0/40)	36.6	(15/41)	37.5	(15/40)	37.5	(15/40)	46.3	(19/41)	32.5	(13/40)
**3**	83.3	(25/30)	13.3*	(4/30)	70	(21/30)	70	(7/10)	73.3	(22/30)	83.3	(25/30)	90	(9/10)
**4**	86.7	(26/30)	20.7*	(6/29)	59.1	(13/22)	85.7	(12/14)	69	(20/29)	90.9	(20/22)	78.6	(11/14)
**5**	53.3	(16/30)	0.0*	(0/30)	39.3	(11/28)	54.5	(12/22)	66.7	(20/30)	50	(14/28)	40.9	(9/22)
**6**	70	(21/30)	10.0*	(3/30)	43.3	(13/30)	50	(12/24)	70	(21/30)	70	(21/30)	58.3	(14/24)
**7**	66.7	(20/30)	4.9*	(2/41)	71	(22/31)	79.2	(19/24)	68.3	(28/41)	96.8*	(30/31)	95.5*	(21/22)
**8**	33.3	(10/30)	0.0*	(0/41)	36.4	(12/33)	63.6*	(28/44)	58.5	(24/41)	54.5	(18/33)	68.2*	(30/44)
**9**	50	(15/30)	nd		43.3	(13/30)	87.5*	(14/16)	nd		70	(21/30)	62.5	(10/16)

**n/N** Number of mosquitoes positive (Microscopy/ELISA/PCR)/total sample size.

**PI** Post-infection.

*Significant difference in prevalence compared with oocyst prevalence at day 7 PI (P-value <0.05, Chi-squared test).

**nd** Not done.

Colours are demonstrative of relative differences in prevalence between all time points and methods (dark red = High prevalence, light red = low prevalence).
