# Supplementary material for: The relevance and applicability of oocyst prevalence as a read-out for mosquito feeding assays
Source: Sci Rep. 2013 Dec 4;3:3418. doi: 10.1038/srep03418 (PMC4894383; doi:10.1038/srep03418)
Supplement: Supplementary Information [file srep03418-s1.pdf]

Supplementary information:

The relevance and applicability of oocyst prevalence as a read-out for mosquito feeding assays

Will J.R. Stone[1^], Maarten Eldering[1^], Geert-Jan van Gemert[1], Kjerstin H. W. Lanke[1], Lynn Grignard[2], Marga G. van de Vegte-Bolmer[1], Rianne Siebelink-Stoter[1], Wouter Graumans[1], Will F. G. Roeffen[1], Chris J. Drakeley[2], Robert W. Sauerwein[1], Teun Bousema[1,2*]

[1]Department of Medical Microbiology, Radboud university medical center, Nijmegen, The Netherlands

[2]Department of Immunology and Infection, London School of Hygiene and Tropical Medicine, London, United Kingdom

[^]These authors contributed equally

[*]Corresponding author

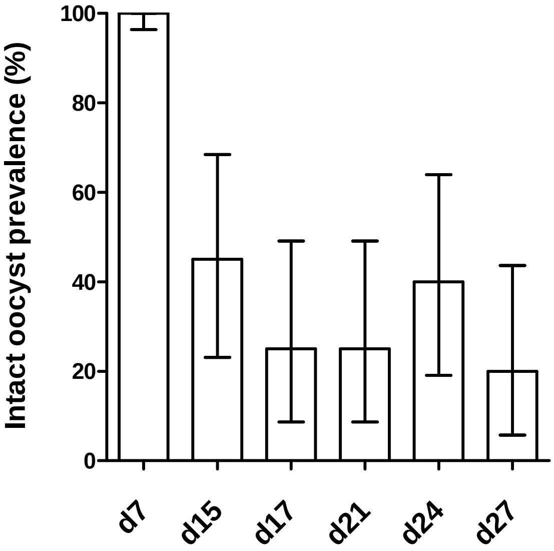

Figure S1. The prevalence of midgut oocysts in mosquito groups from the same experimental feed examined by standard dissection between days 7 and 27 PI. Oocyst prevalence was determined by microscopical examination of mercurochrome stained midguts at all time points (n=20 per time point, n=120 in total). Typically 10-15 mosquitoes per group are dissected as an immediate measure of oocyst prevalence and mean intensity during the SMFA. Oocysts identified here are likely to have been intact, though the possibility that some may have undergone various degrees of rupture or degradation cannot be excluded. Error bars represent 95% confidence intervals (CI). On day 7, median oocyst intensity was 2 (IQR 1-2, range 1-6).

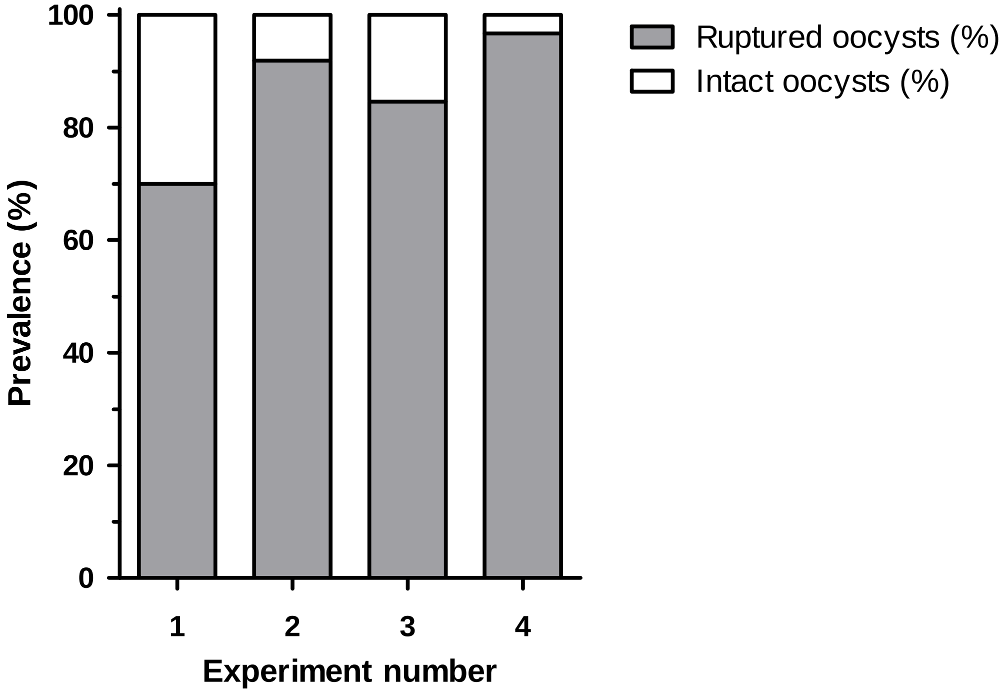

Figure S2. The prevalence of oocyst rupture in infected mosquitoes. Prevalence of oocyst rupture is given as the proportion of mosquitoes in which any oocysts were observed to have undergone rupture. Prevalence of intact oocysts is given as the proportion of mosquitoes in which only intact or degenerated oocysts were observed. All oocyst examination time-points have been combined within experiments, so that experiments 1 and 2 represent the prevalence of oocyst rupture in mosquitoes examined on day 14 and 21 PI, and experiment 3 and 4 the prevalence of oocyst rupture in mosquitoes examined between 11 and 16 days PI. Details of sample numbers are in the text, and in the legend for Fig. 3. Total prevalence of oocyst rupture was 70% (95% CI 53.5-83.4%) in experiment 1, and 91.9% (95% CI 82.2-97.3%) in experiment 2.

Table S1. The results of nine experimental feeds where oocyst prevalence was determined in a sample of mosquitoes at day 7 PI, and infection prevalence was subsequently determined in separate samples collected 7, 10 and 14 days PI by both CSP ELISA and 18s PCR.

		Microscopy			CSP ELISA									18s PCR					
		Mean oocyst intensity	Prevalence % (n/N)		Mean OD	Prevalence % (n/N)		Mean OD	Prevalence % (n/N)		Mean OD	Prevalence % (n/N)		Prevalence % (n/N)					
		Day 7 P1			*Day 7 PI*			*Day 10 PI*			*Day 14 PI*			*Day 7 PI*		*Day 10 PI*		*Day 14 PI*	
**Exp. ID**	**Dilution**																		
**1**	1:5	2.2	**63.3**	(19/30)	0.1	2.5*	(1/40)	0.1	17.5*	(7/40)	0.9	53.8	(21/39)	66.7	(26/39)	60	(24/40)	64.1	(25/39)
**2**	1:10	0.6	**36.6**	(11/30)	0.1	0.0*	(0/40)	0.2	36.6	(15/41)	0.7	37.5	(15/40)	37.5	(15/40)	46.3	(19/41)	32.5	(13/40)
**3**	1:10	4.5	**83.5**	(25/30)	0.1	13.3*	(4/30)	0.7	70	(21/30)	1.5	70	(7/10)	73.3	(22/30)	83.3	(25/30)	90	(9/10)
**4**	1:10	4.7	**86.5**	(25/29)	0.1	20.7*	(6/29)	1.0	59.1	(13/22)	1.4	85.7	(12/14)	69	(20/29)	90.9	(20/22)	78.6	(11/14)
**5**	1:20	1.8	**53**	(16/30)	0.1	0.0*	(0/30)	0.3	39.3	(11/28)	1.0	54.5	(12/22)	66.7	(20/30)	50	(14/28)	40.9	(9/22)
**6**	1:20	2.7	**69**	(21/30)	0.1	10.0*	(3/30)	0.2	43.3	(13/30)	0.9	50	(12/24)	70	(21/30)	70	(21/30)	58.3	(14/24)
**7**	1:10	2.4	**66.5**	(20/30)	0.1	4.9*	(2/41)	0.5	71	(22/31)	1.0	79.2	(19/24)	68.3	(28/41)	96.8*	(30/31)	95.5*	(21/22)
**8**	1:20	0.9	**33**	(10/30)	0.1	0.0*	(0/41)	0.3	36.4	(12/33)	0.7	63.6*	(28/44)	58.5	(24/41)	54.5	(18/33)	68.2*	(30/44)
**9**	1:10	1.3	**50**	(15/30)		nd		0.3	43.3	(13/30)	1.4	87.5*	(14/16)	nd		70	(21/30)	62.5	(10/16)

n/N Number of mosquitoes positive (Microscopy/ELISA/PCR) / total sample size

PI Post-infection

***** Significant difference in prevalence compared with oocyst prevalence at day 7 PI (P-value <0.05, Chi-squared test)

nd Not done

OD Optical density (450nm)

Colours are demonstrative of relative differences in prevalence between all time points and methods (dark red = high prevalence, light red = low prevalence).
